# Combined bone scintigraphy and fluorocholine PET/computed tomography predicts response to radium-223 therapy in patients with prostate cancer

**DOI:** 10.2144/fsoa-2021-0053

**Published:** 2021-05-21

**Authors:** Michele Klain, Valeria Gaudieri, Mario Petretta, Emilia Zampella, Giovanni Storto, Carmela Nappi, Carlo Buonerba, Felice Crocetto, Rosj Gallicchio, Fabio Volpe, Leonardo Pace, Martin Schlumberger, Alberto Cuocolo

**Affiliations:** 1 Department of Advanced Biomedical Sciences, University of Naples Federico II, Naples, Italy; 2 IRCCS SDN, Diagnostic Imaging, Naples, Italy; 3 Nuclear Medicine Unit, IRCCS, Referral Cancer Center of Basilicata, Rionero in Vulture, Italy; 4 Department of Oncology & Hematology, Regional Reference Center for Rare Tumors, AOU Federico II of Naples, Naples, Italy; 5 Department of Neurosciences, Human Reproduction & Odontostomatology, University of Naples Federico II, Naples, Italy; 6 Department of Medicine, Surgery & Dentistry, University of Salerno, Salerno, Italy

**Keywords:** ^18^F-fluorocholine PET/CT outcome, ^223^radium, bone scintigraphy, prostate cancer

## Abstract

**Aim:**

To assess the value of bone scintigraphy and ^18^F-fluorocholine PET/computed tomography (CT) in predicting outcome in patients with prostate cancer and bone metastases treated with ^223^radium.

**Materials & methods:**

Retrospective analysis of 48 patients that underwent ^223^radium therapy. End points were pain relief and overall survival.

**Results:**

After therapy, pain relief was observed in 27 patients. Patients without pain relief had more bone lesions at PET/CT than at bone scintigraphy (pretherapy imaging mismatch). In 39 patients who completed treatment protocol, post-therapy alkaline phosphatase and pretherapy imaging mismatch were independent predictors of poor overall survival.

**Conclusion:**

Patients with more lesions at ^18^F-fluorocholine PET/CT than at bone scintigraphy had a poor prognosis. The combined imaging approach could be useful to predict outcome after ^223^radium therapy.

## Lay abstract

Using a retrospective, two-center cohort of men with prostate cancer (n = 48), this study evaluated the combined assessment of bone scintigraphy and ^18^F-fluorocholine PET/CT in predicting response to ^223^radium. Patients with more bone lesions detected by ^18^F-fluorocholine PET/CT than bone scintigraphy had less symptomatic benefit and the worst outcome.

Worldwide, prostate cancer is the second most common cancer and the sixth leading cause of cancer-related death among men [1]. Bone metastases are common in patients with prostate cancer and are found in around 70% of patients in postmortem studies [2]. Bone metastases are associated with a poor quality of life and with a significant morbidity including pain, pathological fractures and cord compression. The median survival for men with bone metastatic castration-resistant prostate cancer (mCRPC) is approximately 20 months. Several drugs are currently available in clinical practice for the treatment of mCRPC, such as abiraterone acetate, enzalutamide, docetaxel and cabazitaxel [3–5]. Among palliative therapies, radiopharmaceuticals labeled with β-emitters could be used, such as ^89^Sr-chloride, ^153^Sm-ethylene diamine tetra methylene phosphonate, ^188^Re-hydroxyethylidene diphosphonate and ^186^Re-hydroxyethylidene diphosphonate [6]. In the past few years, ^223^radium dichloride, a targeted alpha therapy, became available for the treatment of bone metastases from mCRPC and some clinical variables that may predict tumor response and survival have been identified, such as baseline alkaline phosphatase (ALP), baseline hemoglobin and performance status [7–12].

Nuclear imaging modalities, including bone scintigraphy and PET/computed tomography (PET/CT) are used in these patients [9,13–17]. In particular, PET/CT with choline analogues is highly sensitive in the identification of tumor recurrence [18] and ^11^C-choline PET/CT has been reported to be a predictor of disease-free survival and overall survival (OS) [19]. Despite bone scintigraphy plays an important role in the diagnostic process of patients with prostate cancer, it has low sensitivity in patients with values of prostate-specific antigen (PSA) <20 ng/ml and relatively low specificity [20]. ^18^F-fluorocholine PET/CT has shown to have good sensitivity and specificity in the evaluation of bone metastases [21], with the advantage of detecting nodal and visceral involvement. It has also been reported that in patients with prostate cancer the extent of bone disease and the intensity of ^18^F-fluorocholine uptake are associated with a poor outcome [15]. The aim of the present study was to assess the predictive value of bone scintigraphy and ^18^F-fluorocholine PET/CT for predicting response to treatment with ^223^radium of bone metastases from castration-resistant prostate cancer.

## Materials & methods

### Participants & study design

We retrospectively reviewed the records of 57 consecutive male patients affected by mCRPC who underwent ^223^radium treatment at two different centers, from October 2015 to December 2017. Follow-up ended in February 2020. For the purpose of the present investigation, nine patients for whom both bone scintigraphy and ^18^F-fluorocholine PET/CT data were not available were excluded. Therefore, the final study population comprised 48 patients. All patients had mCRPC with symptomatic bone disease and absence of known visceral and nodal metastases. Patients were treated with ^223^radium (55 kBq/kg, intravenously [iv.]) at a 4-week interval for six cycles, according to approved dose regimen. Treatment completion was defined as patients who had completed six cycles of ^223^radium, whereas a premature discontinuation of treatment protocol because of clinical or radiological progression or for a grade 4 adverse event was considered as treatment failure. The patients were maintained on androgen deprivation therapy. For each patient baseline characteristics, previous treatments and patient outcome were recorded. Pain intensity was scored using a 5-point scale: 0 (no pain); 1 (mifld pain); 2 (moderate pain); 3 (severe pain); and 4 (incoercible pain) [22]. Each patient was re-evaluated before each ^223^radium administration and 1 month after the last ^223^radium administration. Blood tests were collected at baseline, before each cycle and at the end of ^223^radium therapy, including white blood cell count, hemoglobin, platelet count, alkaline phosphatase (ALP) and prostate-specific antigen (PSA). Safety was assessed on the basis of hematologic and gastrointestinal adverse events and limiting normal physical activity. The date of the last consultation or of death was used to determine the length of follow-up.

### Imaging techniques

Bone scintigraphy and ^18^F-fluorocholine PET/CT were performed within 1 month before the first administration of ^223^radium, and within 2 weeks from each other. Whole body bone scintigraphy was acquired 3 h after iv. injection of 740 MBq of ^99m^Tc-methylene-diphosphonate (MDP), using a dual-head gamma camera (E.CAM, Siemens Medical Systems, IL, USA) equipped with low energy, high-resolution collimators. ^18^F-fluorocholine PET/CT was performed 45–60 min after iv. administration of 2–4 MBq/kg, in 3D acquisition mode for 3–8 min per bed position, from skull to feet, using a Discovery PET/CT (GE Healthcare, WI, USA). Low-dose CT (120 kV, 80 mA) was performed for attenuation correction and as an anatomical map. No contrast was injected. Criteria to define bone metastases included the presence of a focal area of increased tracer uptake higher than background, with or without any underlying lesion identified using CT and outside of joint location or osteophytes or excretion locations.

Two independent observers for each center evaluated bone scintigraphy and ^18^F-fluorocholine PET/CT. In case of disagreement, a third observer reviewed the studies to reach a consensus. Imaging findings were used to assess tumor burden. Based on the number of bone lesions, patients were divided into three groups: group 1, less than six lesions; group 2, from six to 20 lesions; and group 3 more than 20 lesions [23]. Based on the number of lesions detected with each method, patients were categorized as showing more lesions on ^18^F-fluorocholine PET/CT scan than on bone scintigraphy, an equal number of lesions or less lesions on ^18^F-fluorocholine PET/CT scan than on bone scintigraphy.

### Outcome

The clinical end points were pain course and OS. Pain relief was defined as a reduction in pain category by comparing pain categories before and after therapy. OS was defined as the elapsed time between the date of the first injection of ^223^radium and the date of death and patients who were still alive at last follow-up were censored on that date.

### Statistical analysis

Continuous data are expressed as mean ± standard deviation and categorical data as percentage. A student two-sample *t-*test and χ^2^ test were used to compare the differences in continuous and categorical variables, respectively. Lin’s concordance correlation co-efficient and Bland–Altman analysis were used to evaluate the agreement between ^18^F-fluorocholine PET/CT and bone scintigraphy. Univariate and multivariate Cox regression analyses for OS were performed. Event-free survival curves were obtained by the Kaplan–Meier method and compared with the logrank test. Cut-off values for quantitative variables (ALP) were estimated with receiver operating characteristic curve (ROC) analysis using death as gold standard. At ROC analysis, a cut-off value of 208 U/l of post-therapy ALP showed a high sensitivity and a moderate specificity in the prediction of death. This value was used to stratify patients at Kaplan–Meier survival analysis. A p-value < 0.05 was considered statistically significant. MedCalc Statistical Software version 19.2.6 (MedCalc Software Ltd, Ostend, Belgium) was used for statistical analysis.

## Results

At the start of treatment with ^223^radium, the 48 patients included had a median age of 73 years (interquartile range: 68–80 years). The follow-up was 15 ± 10 months (range: 2–43 months). Thirty-four patients died during follow-up. The six-cycle treatment was completed for 39 (81%) patients. Twenty-one (44%) patients had an anemia and five (10%) a thrombocytopenia, of whom one patient discontinued therapy for grade 4 thrombocytopenia, seven (15%) reported nausea and 14 (30%) fatigue. Treatment was discontinued prematurely in nine (19%) patients. The reason for treatment discontinuation was grade 4 thrombocytopenia (in one patient), disease-related death (in four patients), and disease progression (in four patients).

**Figure 1.**
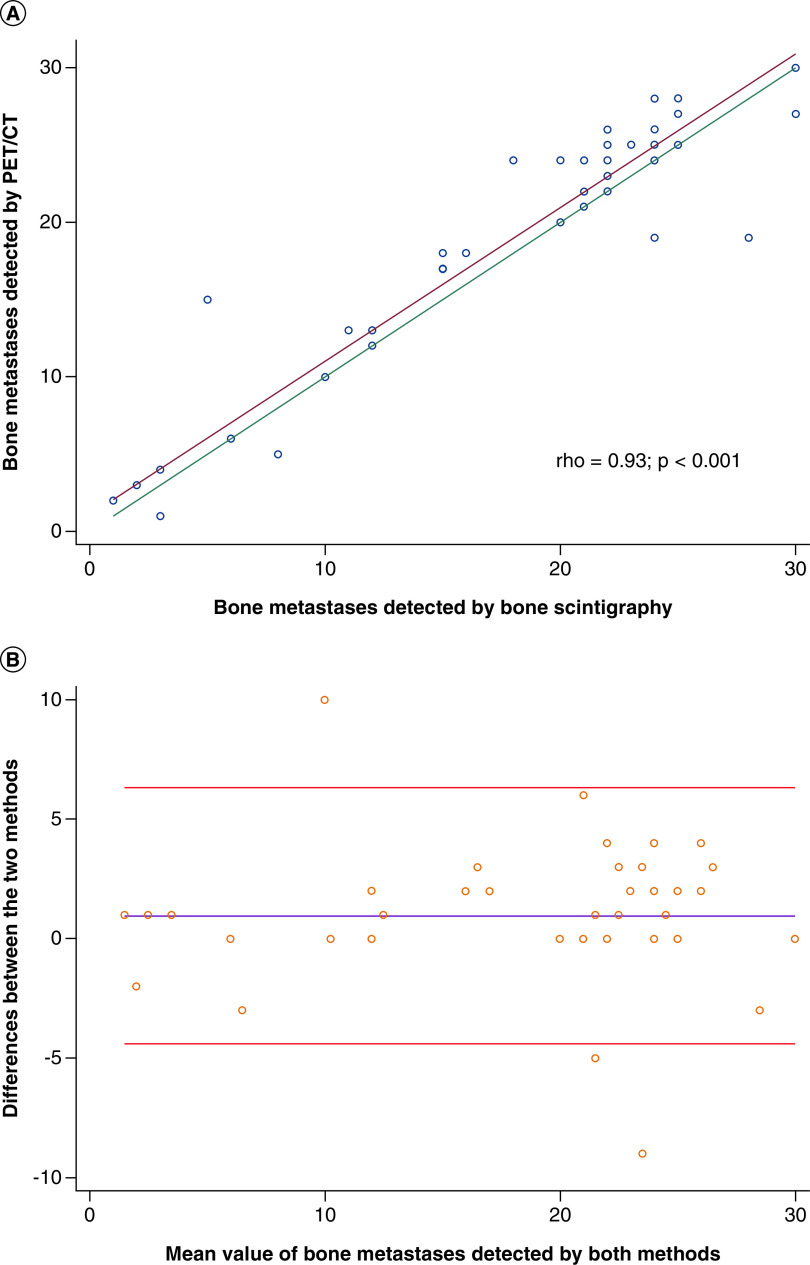
Agreement between bone scintigraphy and ^18^F-fluorocholine PET/CT in the identification of bone metastases. **(A)** Scatter plots of bone metastases detected by bone scintigraphy and ^18^F-fluorocholine PET/CT. Red line indicates the reduced major axis, green line perfect concordance and Lin’s concordance correlation co-efficient. **(B)** Bland–Altman analysis. The mean difference (95% limits of agreement) is 0.958 (-4.405 to 6.321). CT: Computed tomography.

The scatter plot of bone metastases detected by bone scintigraphy and ^18^F-fluorocholine PET/CT and Bland–Altman analysis are shown in Figure 1. The Lin’s co-efficient value was excellent (>0.90). However, at Bland–Altman analysis the bias was 0.958 with 95% limits of agreement from -4.405 to 6.321 and three patients were outlier.

**Figure 2.**
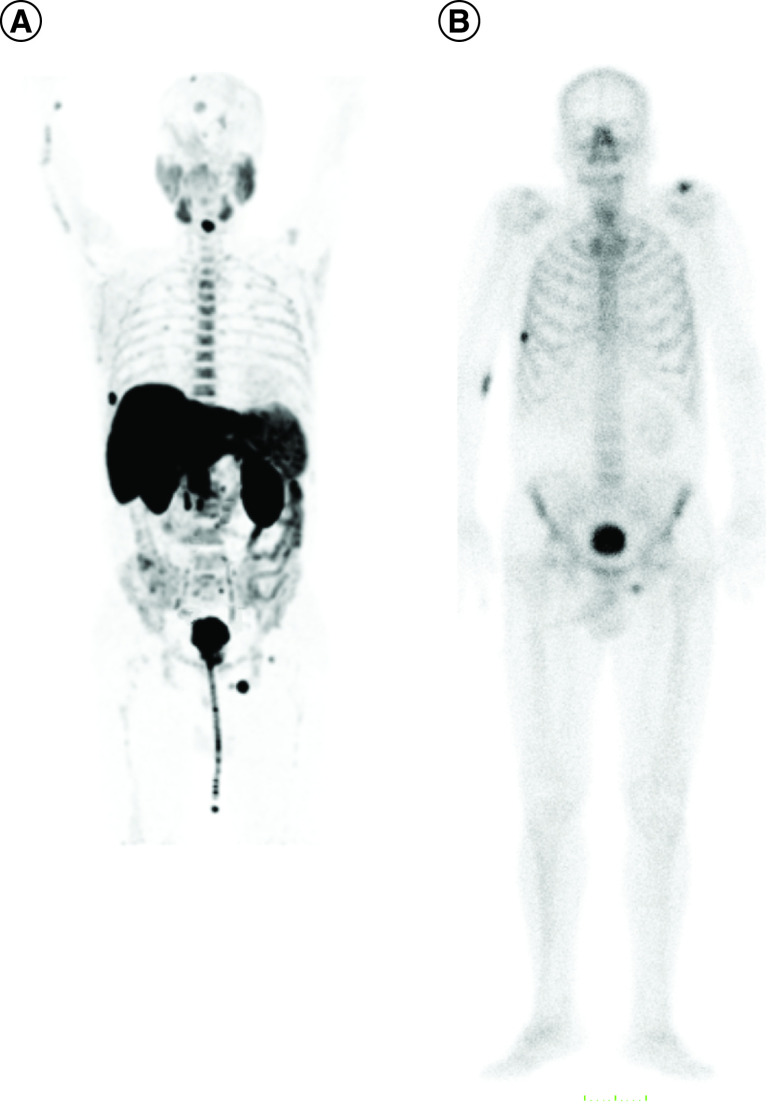
Example of a patient with baseline imaging mismatch. **(A)**^18^F-fluorocholine PET/CT. **(B)** Bone scintigraphy. The number of bone lesions detected by PET/CT is higher than that detected by bone scintigraphy. CT: Computed tomography.

Even if there was an excellent correlation between the two methods, only in 18 patients bone scintigraphy and ^18^F-fluorocholine PET/CT detected the same number of bone metastases. In 25 patients ^18^F-fluorocholine PET/CT identified more metastases than bone scintigraphy and in the remaining five patients bone scintigraphy found more metastases than ^18^F-fluorocholine PET/CT (p < 0.05). Figure 2 illustrates an example of a patient with more bone metastases at ^18^F-fluorocholine PET/CT compared with bone scintigraphy.

**Figure 3.**
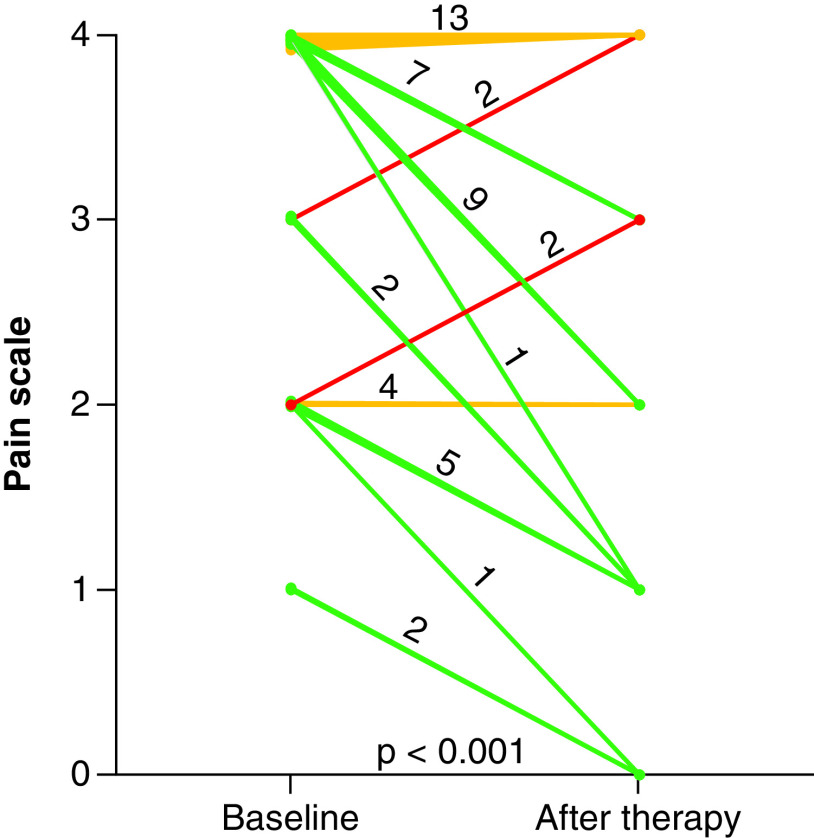
Pain intensity variation from baseline to post-treatment. After ^223^radium therapy, there is a significant (p < 0.001) change in pain intensity, with pain relief in 27 (56%) patients.

After ^223^radium therapy, 27 patients (56%) had pain relief, 17 (35%) no variation in pain intensity and four (8%) pain exacerbation (Figure 3). Table 1 shows clinical characteristics, biochemical data and imaging findings according to pain relief status. More bone lesions were seen at ^18^F-fluorocholine PET/CT than at bone scintigraphy in 15 of the 21 (71%) patients without pain relief and in ten (37%) of 27 patients with pain relief (p < 0.05). Patients without pain relief had a higher prevalence of treatment failure compared with those with pain relief (33 vs 7%; p < 0.005).

**Table 1.** Clinical characteristics, biochemical data and imaging findings according to pain relief status.

	All patients (n = 48)	Pain relief (n = 27)	No pain relief (n = 21)	p-value
Age (years)	73 ± 9	71 ± 8	75 ± 9	0.2
Baseline PSA (ng/ml)	836 ± 3961	1165 ± 5265	411 ± 601	0.5
Post-therapy PSA (ng/ml)	1174 ± 2394	1045 ± 2507	1341 ± 2292	0.7
Baseline ALP (U/l)	417 ± 698	294 ± 444	577 ± 920	0.2
Post-therapy ALP (U/l)	307 ± 331	217 ± 222	425 ± 411	<0.05
Baseline Hb (g/dl)	12 ± 1.6	12 ± 1.8	12 ± 1.3	0.7
Post-therapy Hb (g/dl)	11 ± 1.5	11.1 ± 1.6	10 ± 1.3	0.1
Baseline WBC (×10^9^/l)	6.4 ± 2.2	6.5 ± 1.8	6.1 ± 2.2	0.5
Post-therapy WBC (×10^9^/l)	5 ± 1.5	5 ± 1.1	5.5 ± 1.6	0.1
Baseline PLT (×10^9^/l)	231 ± 80	231 ± 92	223 ± 60	0.8
Post-therapy PLT (×10^9^/l)	192 ± 88	190 ± 71	194 ± 107	0.9
Gleason score	7.9 ± 1	7.8 ± 1	8.1 ± 1	0.5
Pain intensity category	4 (1–4)	4 (1–4)	4 (2–4)	0.05
Treatment failure, n (%)	9 (19%)	2 (7%)	7 (33%)	<0.005
Baseline bone scintigraphy				
Bone metastases (n)	19 ± 8	19 ± 8	19 ± 7	0.8
Extent of metastases, n (%) <6 lesions 6–20 lesions >20 lesions	5 (10%) 11 (23%) 32 (67%)	3 (11%) 4 (15%) 20 (74%)	2 (10%) 7 (33%) 12 (57%)	0.3
Baseline PET/CT				
Bone metastases (n)	18 ± 9	20 ± 8	20 ± 7	0.9
Extent of metastases, n (%) <6 lesions 6–20 lesions >20 lesions	5 (10%) 12 (25%) 31 (65%)	3 (11%) 5 (19%) 19 (70%)	2 (10%) 7 (33%) 12 (57%)	0.5

Pain intensity category is expressed as median value (range), other values are expressed as mean value ± standard deviation or as number (percentage).

ALP: Alkaline phosphatase; CT: Computed tomography; Hb: Hemoglobin; PLT: Platelet count; PSA: Prostate-specific antigen; WBC: White blood cell count.

At univariate analysis, variables associated with a poor OS were: high Gleason score, ^223^radium treatment failure, high baseline ALP level, high post-therapy ALP level, persistent or worsened post-therapy pain and pretherapy imaging mismatch with more lesions seen on PET/CT than on bone scintigraphy (Table 2). At multivariate analysis, only treatment failure and high post-therapy ALP level were independent predictors of a poor OS (both p < 0.05).

**Table 2.** Univariate Cox regression analysis for overall survival in the 48 patients under study.

	Chi-square	p-value
Age	0.69	0.4
Gleason score	4.90	<0.05
Treatment failure	41.1	<0.001
High baseline PSA	0.03	0.87
High post-therapy PSA	0.07	0.78
High baseline ALP	2.96	<0.05
High post-therapy ALP	8.91	<0.001
Baseline pain	1.48	0.24
Post-therapy pain	4.59	<0.05
Number of bone metastases at bone scintigraphy	2.18	0.15
Extent of bone metastases at bone scintigraphy	0.58	0.46
Number of bone metastases at PET/CT	1.83	0.19
Extent of bone metastases at PET/CT	0.77	0.39
Baseline imaging mismatch^†^	5.62	<0.02

† More lesions on PET/CT than bone scintigraphy.

ALP: Alkaline phosphatase; CT: Computed tomography; PSA: Prostate-specific antigen.

In consideration of the strong association between treatment failure and poor outcome, we performed a subanalysis restricted to the 39 patients who completed the six-cycle treatment (Table 3). High baseline and post-therapy ALP levels, persistent or worsened post-therapy pain and pretherapy imaging mismatch with more lesions seen on PET/CT than on bone scintigraphy were predictors of a poor OS. At multivariate analysis, only high post-therapy ALP level and pretherapy imaging mismatch remained significantly associated with a poor OS (both p < 0.05).

**Table 3.** Univariate Cox regression analysis for overall survival in the 39 patients who completed the six-cycle treatment.

	Chi-square	p-value
Age	1.0	0.3
Gleason score	0.10	0.7
High baseline PSA	0.03	0.8
High post-therapy PSA	0.01	0.9
High baseline ALP	4.01	<0.05
High post-therapy ALP	10.13	<0.002
Baseline pain	2.40	0.1
Post-therapy pain	5.61	<0.05
Number of bone metastases at bone scintigraphy	0.34	0.5
Extent of bone metastases at bone scintigraphy	0.70	0.4
Number of bone metastases at PET/CT	2.76	0.1
Extent of bone metastases at PET/CT	2.2	0.1
Baseline imaging mismatch^†^	8.62	<0.005

† More lesions on PET/CT than bone scintigraphy.

ALP: Alkaline phosphatase; CT: Computed tomography; PSA: Prostate-specific antigen.

**Figure 4.**
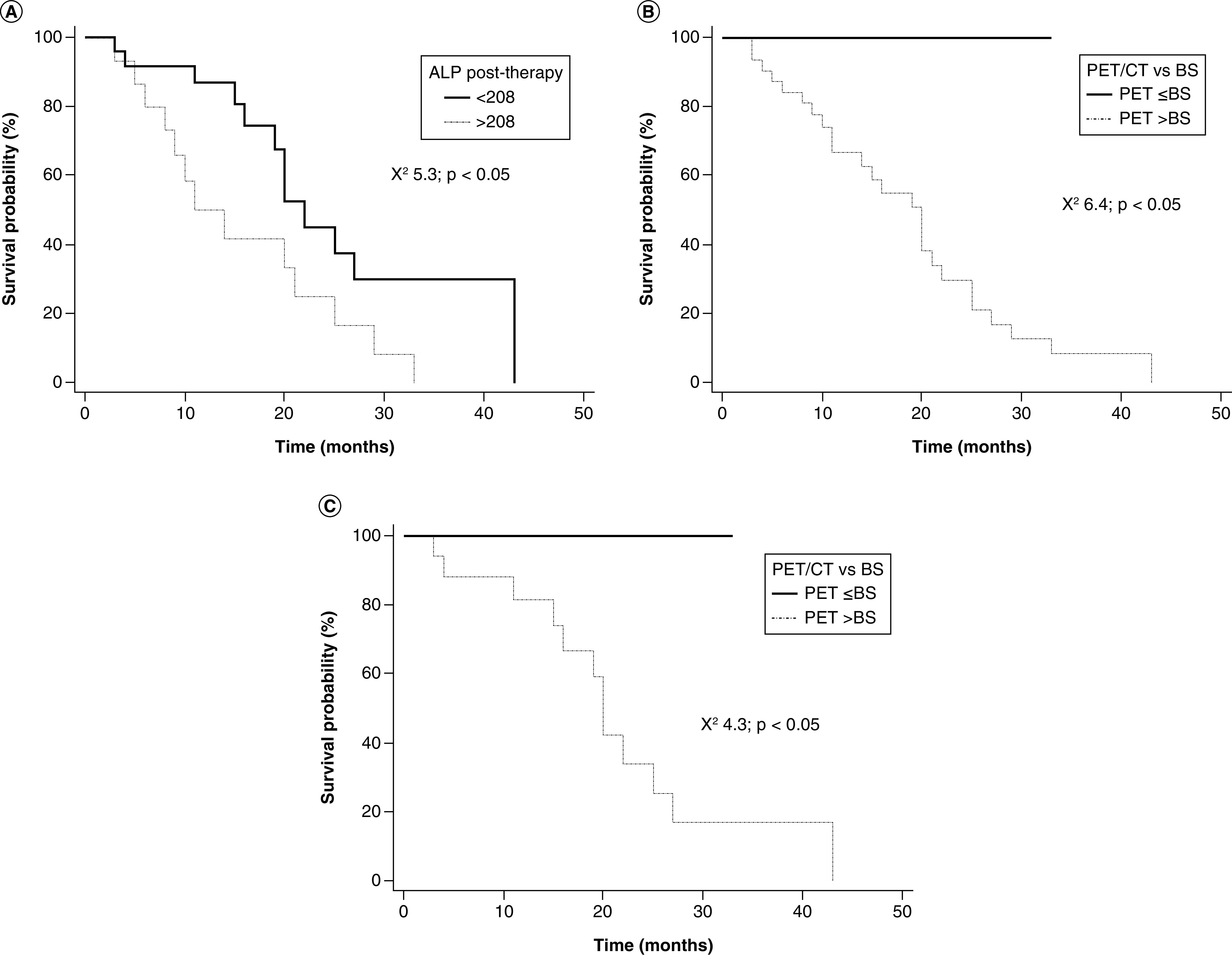
Overall survival curves after ^223^radium therapy. **(A)** Patients stratified by post-therapy ALP level (≤208 vs >208 U/l). **(B)** Patients stratified by imaging findings (PET/CT vs bone scintigraphy). **(C)** Patients with post-therapy ALP level ≤208 U/l stratified by imaging findings. Patients with post-therapy ALP >208 U/l level have the worst outcome. Imaging mismatch (number of bone lesions detected by PET/CT >number of bone lesions detected by bone scintigraphy) predicts death in the overall population and in patients with post-therapy ALP levels ≤208 U/l. ALP: Alkaline phosphatase; BS: Bone scintigraphy; CT: Computed tomography.

Figure 4 illustrates the survival curves in the 39 patients who completed the six-cycle treatment stratified by post-therapy ALP level and imaging results. Patients with post-therapy ALP level >208 U/l had the worst outcome. Mismatch between bone scintigraphy and ^18^F-fluorocholine PET/CT (number of bone lesions detected by PET/CT >number of bone lesions detected by bone scintigraphy) predicted death in the overall population and in patients with post-therapy ALP level ≤208 U/l.

## Discussion

This study first demonstrates that the presence of more bone lesions detected by ^18^F-fluorocholine PET/CT compared with bone scintigraphy is associated with poor prognosis in patients with mCRPC treated with ^223^radium.

No previous reports exist concerning to the use of combined assessment of baseline bone scintigraphy and ^18^F-fluorocholine PET/CT in the outcome prediction with ^223^radium treatment. Moreover, data on the use of ^18^F-choline PET/CT in the evaluation of ^223^radium treatment efficacy are limited. Some studies reported that baseline ^18^F-fluorocholine PET/CT is a prognostic indicator for both OS and risk of a skeletal-related event [12,15]. García Vicente *et al.* [15] studied 40 patients treated with ^223^radium. They found that extensive disease on bone scintigraphy or ^18^F-fluorocholine PET/CT, high intensity of ^18^F-fluorocholine uptake and a baseline PSA serum level above 58.3 ng/ml were associated with unfavorable OS. No clinical or imaging variable was able to predict the progression-free survival, probably due to the high rate of progressive disease. Twenty patients did not complete all six cycles due to progressive disease and treatment failure was an independent predictor for OS. Differently, considering that therapeutic failure has a strong negative prognostic impact, we also performed a subanalysis restricted to patients who successfully completed the six treatment cycles. In these patients, we found that mismatch between bone scintigraphy and ^18^F-fluorocholine PET/CT with more bone metastases detected by ^18^F-fluorocholine PET/CT was associated with worst outcome.

Choline is a component of phosphatidylcholine, an essential element of phospholipids in the cell membrane [24]. ^18^F-choline closely mimics choline uptake by normal tissue and prostate cancer cells. A nonsignificant relationship between choline uptake and abnormal PSA serum level has been reported [25]. However, serum PSA values do not strictly correlate with the size or number of tumor lesions [26]. Higher-grade tumor cells produce less PSA and advanced-stage tumors contain high-grade tumor cells [27]. There is no linear correlation between PSA and ALP values and PSA is inferior to ALP in the stratification of the metastatic bone burden [27]. Serum ALP level is a prognostic biomarker in mCRPC, predicting disease outcome independently of therapy and PSA values and high baseline ALP values are related with negative outcomes, including skeletal complications and decreased survival [28]. One study reported two patients who underwent ^223^radium therapy and were evaluated with ^18^F-fluorocholine PET/CT, one patient, demonstrating a near complete resolution of abnormal PET activity, had a normalization of his serum ALP level, while the other patient, demonstrating heterogeneous changes on PET, continued to have significantly elevated levels of ALP [29].

Ceci *et al.* [30] reported a potential role of ^11^C-choline PET in the identification of patients treated with docetaxel with radiological progression despite a PSA response and found that a higher number of lesions at baseline PET is associated with an increased probability of progressive disease. The evaluation of the burden of bone metastases is essential before the use of ^223^radium [10,31] and according to the ALSYMPCA trial ^223^radium treatment is more effective in patients with a moderate burden of disease [23]. It has been demonstrated that the extent of bone disease on the bone scan correlates with survival and patients with less than six lesions had the best outcome [32].

In our study, the treatment was well tolerated and the majority of adverse events were grade 1 or 2. Only 9/48 patients did not complete all six treatment cycles. Regarding imaging findings, in many patients ^18^F-fluorocholine PET/CT was able to detect more bone metastases than bone scintigraphy and this is not an unexpected result in consideration of the better spatial resolution of PET/CT, which increases its sensitivity; however, we found an excellent correlation between the two methods. No imaging variable was able to predict OS, but we found that patients with more lesions detected by ^18^F-fluorocholine PET/CT compared with bone scintigraphy had the worst OS and these patients seem to have less symptomatic benefit derived from ^223^radium therapy. From a pathophysiological point of view, it could be ascribed to the different mechanism of tracers uptake since fluorocholine is a cell membrane turn-over indicator and diphosphonates adsorption better reflects bone mineral microenvironment. Moreover, ^18^F-fluorocholine PET/CT is able to detect bone marrow metastases without significant bone reaction and remodeling [21], allowing an earlier detection of bone metastases compared with bone scintigraphy. On the other hand, bone metastases detected by MDP are more sensitive to ^223^radium effect compared with bone metastases that show no MDP uptake, probably because MDP uptake predicts ^223^radium uptake in bone metastases by a similar mechanism. This is in accordance with the poor effect of ^223^radium in osteolytic lesions (MDP negative) observed in thyroid cancer patients [33] and could explain the worse outcome of patients with more lesions detected by ^18^F-fluorocholine PET/CT compared with bone scintigraphy. Regarding patients’ reported pain, consistently with previous real-world data [34], 56 and 35% of patients reported an improvement and a stabilization of pain at the end of the treatment, respectively. Patients who did not have pain relief had a significantly higher prevalence of pretherapy mismatch between imaging methods with more lesions detected at PET/CT, confirming that ^223^radium accumulates in osteoblastic metastases and exerts its therapeutic action on these lesions.

Moreover, patients with lower ALP values after therapy had a better OS. This finding is in agreement with other studies [35] and high ALP values are an expression of a more extensive disease burden, which carries a shorter life expectancy and a reduced chance for response. We observed that even in patients with lower values of ALP, imaging mismatch allows the identification of those who have a worse prognosis.

Our study supports the concept that the existing selection criteria for ^223^radium treatment are insufficient. It is important to know pretherapeutic prognostic factors to achieve better patient selection for ^223^radium treatment, also reducing healthcare costs. The impact of the extent of skeletal disease on OS was already investigated in a retrospective study demonstrating that quantitative analysis of bone tumor burden, assessed by baseline ^18^F-fluoride PET/CT, was a predictor of OS [16]. Murray *et al.* [13] found in a small group of patients a strong relationship between ^18^F-fluoride uptake and ^223^radium absorbed dose and the subsequent lesion response, indicating that fluoride activity could be a predictor of lesion absorbed dose. Uprimny *et al.* [36] compared the diagnostic performance of ^68^Ga prostate-specific membrane antigen (PSMA) PET/CT and ^18^F-fluoride PET/CT in the assessment of bone metastases in a group of 16 patients. They found that ^18^F-fluoride PET/CT detect more bone lesions than ^68^Ga PSMA. However, ^68^Ga PSMA PET/CT also provided information on visceral involvement suggesting that a combined approach should be preferred. Another study evaluated the role of ^18^F-FDG PET/CT in predicting the response to ^223^radium therapy [37]. The results demonstrated that metabolic tumor volume identified a subgroup of patients with worse prognosis, suggesting a possible role of ^18^F-FDG PET/CT as a tool for the selection of candidates to ^223^radium therapy.

Our study has some limitations. It is a retrospective analysis including a small number of patients. The heterogeneity of the patient population might influence the results and prevents solid conclusions from being drawn. Therefore, prospective trials in larger patient populations are needed. However, we report the experience of two different centers in ^223^radium treatment of patients with mCRPC, avoiding the bias of a single-center study.

## Conclusion

Patients with more bone lesions detected by ^18^F-fluorocholine PET/CT compared with bone scintigraphy have less symptoms improvement and the worst outcome. Our results suggest that this combined approach could help in identifying patients who have the greatest benefits from ^223^radium treatment. Therefore, the combined findings of these techniques could be used as a decision aid to guide the therapeutical management.

## Future perspective

The assessment of the burden of bone metastases is crucial before ^223^radium, since the best results for ^223^radium therapy are observed in patients with a moderate burden of disease. ^68^Ga-PSMA or ^18^F-PSMA could be used in the evaluation of bone metastases and of their response to ^223^radium treatment, considering that PSMA has a higher sensitivity than ^18^F-fluorocholine PET/CT, even with low serum PSA levels. In addition, it is used in the selection of candidates to ^177^Lu labeled PSMA, that represents a promising option in patients with mCRPC and probably will become the future of radioligand therapy.

**Summary points**

- Bone metastasis burden evaluation is essential before ^223^radium therapy.
- Combined bone scintigraphy and choline PET/computed tomography (CT) could improve risk stratification.
- Patients without pain relief had more lesions detected by ^18^F-fluorocholine PET/CT compared with bone scintigraphy and a higher prevalence of treatment failure.
- Patients with more metastases seen by PET/CT had the worst outcome.
- The combined imaging approach could help in identifying patients who have the greatest benefits from ^223^radium treatment.
